# Deep Learning With Spiking Neurons: Opportunities and Challenges

**DOI:** 10.3389/fnins.2018.00774

**Published:** 2018-10-25

**Authors:** Michael Pfeiffer, Thomas Pfeil

**Affiliations:** Bosch Center for Artificial Intelligence, Robert Bosch GmbH, Renningen, Germany

**Keywords:** neural networks, spiking neurons, neuromorphic engineering, event-based computing, deep learning, binary networks

## Abstract

Spiking neural networks (SNNs) are inspired by information processing in biology, where sparse and asynchronous binary signals are communicated and processed in a massively parallel fashion. SNNs on neuromorphic hardware exhibit favorable properties such as low power consumption, fast inference, and event-driven information processing. This makes them interesting candidates for the efficient implementation of deep neural networks, the method of choice for many machine learning tasks. In this review, we address the opportunities that deep spiking networks offer and investigate in detail the challenges associated with training SNNs in a way that makes them competitive with conventional deep learning, but simultaneously allows for efficient mapping to hardware. A wide range of training methods for SNNs is presented, ranging from the conversion of conventional deep networks into SNNs, constrained training before conversion, spiking variants of backpropagation, and biologically motivated variants of STDP. The goal of our review is to define a categorization of SNN training methods, and summarize their advantages and drawbacks. We further discuss relationships between SNNs and binary networks, which are becoming popular for efficient digital hardware implementation. Neuromorphic hardware platforms have great potential to enable deep spiking networks in real-world applications. We compare the suitability of various neuromorphic systems that have been developed over the past years, and investigate potential use cases. Neuromorphic approaches and conventional machine learning should not be considered simply two solutions to the same classes of problems, instead it is possible to identify and exploit their task-specific advantages. Deep SNNs offer great opportunities to work with new types of event-based sensors, exploit temporal codes and local on-chip learning, and we have so far just scratched the surface of realizing these advantages in practical applications.

## 1. Introduction

Training and inference with deep neural networks (DNNs), commonly known as deep learning (LeCun et al., 2015; Schmidhuber, 2015; Goodfellow et al., 2016), has contributed to many of the spectacular success stories of artificial intelligence (AI) in recent years (Goodfellow et al., 2014; Amodei et al., 2016; He et al., 2016; Silver et al., 2016). Models of cortical hierarchies from neuroscience have strongly inspired the architectural principles behind DNNs (Fukushima, 1988; Riesenhuber and Poggio, 1999), but at the implementation level, only marginal similarities between brain-like computation and analog neural networks (ANNs) as used in AI applications can be recognized. One obvious difference is that neurons in ANNs are mostly non-linear but continuous function approximators that operate on a common clock cycle, whereas biological neurons compute with asynchronous *spikes* that signal the occurrence of some characteristic event by digital and temporally precise action potentials. In recent years, researchers from the domains of machine learning, computational neuroscience, neuromorphic engineering, and embedded systems design have tried to bridge the gap between the big success of DNNs in AI applications and the promise of spiking neural networks (SNNs) (Maass, 1997; Ponulak and Kasinski, 2011; Grüning and Bohte, 2014). This promise of SNNs results from their favorable properties exhibited in real neural circuits like brains, such as analog computation, low power consumption, fast inference, event-driven processing, online learning, and massive parallelism. Furthermore, event-based vision and audio sensors (Lichtsteiner et al., 2008; Posch et al., 2014; Liu et al., 2015) have reached an increasingly mature level, and deep SNNs are one of the most promising concepts for processing such inputs efficiently (Tavanaei et al., 2018). This line of research has coincided with an increased interest in efficient hardware implementations for conventional DNNs, since the massive hunger for computational resources has turned out to be a major obstacle as deep learning makes its way toward real-world applications such as automated driving, robotics, or the internet of things (IoT). Concepts such as so-called *binary networks*, which allow in-memory computations, share a binary and potentially sparse communication scheme with SNNs. However, such networks are typically executed in a synchronized manner, which is different from the event-driven (asynchronous) mode of execution in SNNs. Consequently, a fruitful interdisciplinary exchange of ideas to build neuromorphic systems for these concepts is taking place.

In this review, we provide an overview of several key ideas behind deep SNNs, and discuss challenges and limitations of SNNs compared to their ANN counterparts, as well as opportunities for future applications, in particular in conjunction with novel computing models and hardware currently being developed. This article is structured as follows: Section 2 discusses the preparation of input and output in order to perform inference with deep SNNs. In section 3, we give an overview of how deep SNNs can be trained, how this is connected to training conventional DNNs, and how to possibly learn on spike level. Section 4 discusses efficient implementations of deep SNNs on neuromorphic hardware and their limitations, as well as highlights similarities to hardware-efficient solutions for conventional DNNs. In section 5, we present possible use cases of deep SNNs, and argue that their strengths are complementary to those of conventional DNNs. Finally, section 6 provides a discussion of the state-of-the-art, and gives an outlook on promising research directions.

### 1.1. What is a deep spiking neural network?

**Figure 1:**
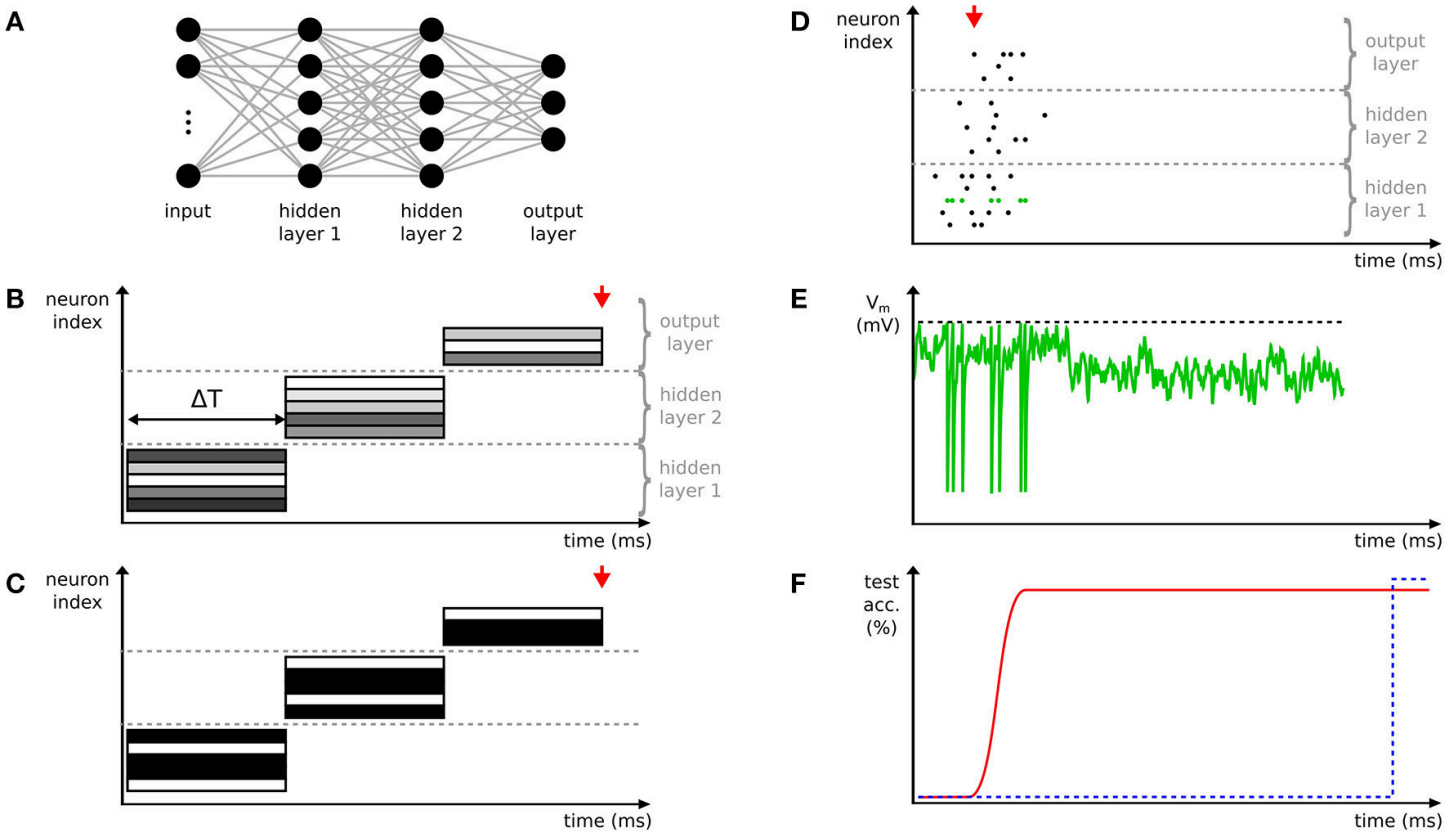
Comparison of deep spiking neural networks (SNNs) to conventional deep neural networks (DNNs). **(A)** Example of a deep network with two hidden layers. Here, exemplarily a fully-connected network is shown. Neurons are depicted with circles, connections with lines. **(B)** Time-stepped layer-by-layer computation of activations in a conventional DNN with step duration Δ*T*. The activation values of neurons (rectangles) are exemplarily visualized with different gray values. The output of the network, e.g. categories in the case of a classification task, is only available after all layers are completely processed. **(C)** Like **(B)**, but with binarized activations. **(D)** The activity of a deep SNN showing a fast and asynchronous propagation of spikes through the layers of the network. **(E)** The membrane potential of the neuron highlighted in green in **(D)**. When the membrane potential (green) crosses the threshold (black dashed line) a spike is emitted and the membrane potential is reset. **(F)** The first spike in the output layer (red arrow in **D**) rapidly estimates the category (assuming a classification task) of the input. The accuracy of this estimation increases over time with the occurrence of more spikes (red line and Diehl et al., 2015). In contrast, the time-stepped synchronous operation mode of DNNs results in later, but potentially more accurate classifications compared to SNNs (blue dashed line and red arrows in **B,C**).

Neural networks are typically called *deep* in case they have at least two hidden layers computing non-linear transformations of the input. In this article, we consider only feed-forward networks, which compute a mapping from input to output (for an example see Figure 1A), and do not address recurrent neural networks. Our definition includes multi-layer fully-connected networks, convolutional neural networks (CNNs; LeCun and Bengio, 1995), deep belief networks (DBNs; Hinton et al., 2006), deep autoencoders, and many more.

Spiking neural networks were originally studied as models of biological information processing (Gerstner and Kistler, 2002), in which neurons exchange information via spikes (for an example, see Figure 1D). It is assumed that all spikes are stereotypical events, and, consequently, the processing of information is reduced to two factors: first, the timing of spikes, e.g., firing frequencies, relative timing of pre- and postsynaptic spikes, and particular firing patterns. Second, the identity of the synapses used, i.e., which neurons are connected, whether the synapse is excitatory or inhibitory, the synaptic strength, and possible short-term plasticity or modulatory effects. Depending on the level of detail of the simulation neurons are either point neurons in which arriving spikes immediately change their (somatic) membrane potentials, or are modeled as multi-compartment models with complex spatial (dendritic) structure, such that dendritic currents can interact before the somatic potential is modified. Different spiking neuron models such as the integrate-and-fire, spike response, or Hodgkin-Huxley model describe the evolution of the membrane potential and spike generation in different levels of detail. Typically, the membrane potential integrates currents from arriving spikes and generates a new spike whenever some threshold is crossed (Figure 1E). Once a spike is generated, it is sent via the axon to all connected neurons with a small axonal delay and the membrane potential is reset toward a given baseline.

The most direct connection between analog and spiking neural networks is made by considering the activation of an analog neuron as the equivalent of the firing rate of a spiking neuron assuming a steady state. Many models of neuronal measurements have used such rate codes to explain computational processes in brains (Hubel and Wiesel, 1959; Rieke, 1999). However, spiking neuron models can also model more complex processes that depend on the relative timing between spikes (Gütig, 2014) or on timing relative to some reference signal, such as network oscillations (Montemurro et al., 2008). Temporal codes are of high importance in biology where even a single spike or small temporal variations of single neuron firing may trigger different reactions (Gerstner et al., 1996; Stemmler, 1996; Rieke, 1999; Machens et al., 2003), because often decisions have to be made before a reliable estimate of a spike rate can be computed.

Besides the biologically motivated definition of SNNs, there is a more pragmatic application-oriented view coming from the field of neuromorphic engineering, where SNNs are often called *event-based* instead of spiking (Liu et al., 2015). Here, an event is a digital packet of information, which is characterized by its origin and destination address, a timestamp, and - in contrast to biologically motivated SNNs—may carry a few bits of payload information. The origin of this view is the address event representation (AER) protocol (Mahowald, 1994; Boahen, 2000), which is used to connect, e.g., event-based sensors (Lichtsteiner et al., 2008) via digital interconnect to neuromorphic chips (Indiveri et al., 2011; Amir et al., 2017) or digital post-processing hardware (Furber et al., 2014). Event-based vision sensors use the payload bits to distinguish visual ON or OFF events, but the payload can also be used to send any other type of relevant information to the postsynaptic targets potentially computing more sophisticated functions than simple integrate-and-fire (Stefanini et al., 2014).

### 1.2. Advantages of deep SNNs

A motivation for studying SNNs is that brains exhibit a remarkable cognitive performance in real-world tasks. With ongoing efforts toward improving our understanding of brain-like computation, there are expectations that models staying closer to biology will also come closer to achieving natural intelligence than more abstract models, or at least will have greater computational efficiency.

SNNs are ideally suited for processing spatio-temporal event-based information from neuromorphic sensors, which are themselves power efficient. The sensors record temporally precise information from the environment and SNNs can utilize efficient temporal codes in their computations as well (Mostafa, 2018). This processing of information is also event-driven meaning that whenever there is little or no information recorded the SNN does not compute much, but when sudden bursts of activity are recorded, the SNN will create more spikes. Under the assumption that typically information from the outside world is sparse, this results in a highly power-efficient way of computing. In addition, using time domain input is additional valuable information compared to frame-driven approaches, where an artificial time step imposed by the sensor is introduced. This can lead to efficient computation of features such as optical flow (Benosman et al., 2014) or stereo disparity (Osswald et al., 2017), and in combination with learning rules sensitive to spike timing leads to more data-efficient training (Panda et al., 2017).

In deep SNNs, the asynchronous data-driven mode of computing leads to fast propagation of salient information through multiple layers of the network. To best exploit this effect in practice, SNNs should be run on neuromorphic hardware. In combination with an event-based sensor, this results in *pseudo-simultaneous* information processing (Farabet et al., 2012; Camuñas-Mesa et al., 2014), which means that a first approximate output of the final layer is available immediately after recording the first input spikes. This is true even for multi-layer networks, because spikes begin to propagate immediately to higher layers as soon as the lower layer provides sufficient activity (Figure 1D). It is not necessary to wait for the complete input sequence to finish, which is in contrast to conventional DNNs, where all layers need to be fully updated before the final output can be computed (Figures 1B,C). The initial output spikes are necessarily based on incomplete information, hence it has been shown that deep SNNs improve their classification performance the longer they are given time to process more spikes of their input (Figure 1F). SNNs can also be trained specifically to reduce the latency of approximate inference (Neil et al., 2016a).

SNNs are the preferred computational model to exploit highly energy-efficient neuromorphic hardware devices, which support the data-driven processing mode, and keep computations local, thereby avoiding expensive memory access operations.

### 1.3. Limitations of deep SNNs

One of the biggest drawbacks of deep SNNs is that despite recent progress (Rueckauer et al., 2017; Sengupta et al., 2018) their accuracy on typical benchmarks such as MNIST (Lecun et al., 1998), CIFAR (Krizehvsky and Hinton, 2009), or ImageNet (Russakovsky et al., 2015) do not reach the same levels as their machine learning counterparts. To some extent, this can be attributed to the nature of these benchmarks, which are on conventional frame-based images. Thus, some form of conversion from images into spike trains is required that is typically lossy and inefficient. Another limiting factor is the lack of training algorithms that make specific use of the capabilities of spiking neurons, e.g., efficient time codes. Instead, most approaches use rate-based approximations of conventional DNNs, which means that no accuracy gains can be expected. Deep SNNs might still be useful in such scenarios, because approximate results might be obtained faster and more efficiently than on conventional systems, especially if the SNN is run on neuromorphic hardware. Training algorithms for SNNs are also more difficult to design and analyze, because of the asynchronous and discontinuous way of computing, which makes a direct application of successful backpropagation techniques as used for DNNs difficult.

The performance of SNNs on conventional AI benchmarks should only be seen as a proof-of-concept, but not as the ultimate research goal. If spiking networks model biology, then we should expect them to be optimized for the behaviorally most relevant tasks, such as making decisions based on continuous input streams while moving in the real world. Image classification corresponds to the task of classifying a random image suddenly flashed on the retina, without any supporting context. While brains are able to solve such tasks (Thorpe et al., 1996), they are certainly not optimized for it. We are currently lacking both good benchmark datasets and evaluation metrics that could measure efficient real-world performance. One fruitful direction is the collection of dynamic vision sensor (DVS) benchmarks (Orchard et al., 2015a; Serrano-Gotarredona and Linares-Barranco, 2015; Hu et al., 2016; Liu et al., 2016), in particular for relevant use cases such as automated driving (Binas et al., 2017; Sironi et al., 2018).

## 2. Inference with deep SNNs

Before diving into the discussion of how to train deep SNNs, we briefly discuss inference with fully trained deep SNNs, i.e., the transformation of input signals to output signals. Whereas updates between hidden layers are straightforward (Gerstner and Kistler, 2002), the input and output layers deserve special attention.

In the ideal case, the input of deep SNNs are already spike trains, e.g., from neuromorphic sensors. However, in many cases, especially when using conventional benchmark datasets, some form of conversion from the input signal into spike trains is necessary. The most widely used method is for each pixel to translate real-valued input such as gray levels or color intensities into spike trains drawn from Poisson processes with proportional firing rates (O'Connor et al., 2013; Cao et al., 2015; Diehl et al., 2015). This implies that only average firing rates are important for classification and information of precise timing is neglected. Although this is clearly a sub-optimal use of SNNs, the method is effective in practice and can be realized in hardware (Neil and Liu, 2014; Stromatias et al., 2015; Schmitt et al., 2017).

Alternative codes that enable an efficient use of spike times have only recently been introduced to spiking deep networks. Kheradpisheh et al. (2018) use a rank-order code in which every neuron can fire at most once. Mostafa et al. (2017a) propose a very sparse and efficient temporal code, in which the output of a neuron is the time of its first spike. Such codes drastically reduce the number of spikes sent through the network, and training can be achieved via backpropagation (Mostafa, 2018) or STDP (Kheradpisheh et al., 2018). Orchard et al. (2015b) used the timing of spikes to determine maxima in pooling operations. Temporal codes are very efficient, fast, and map well to hardware, but they have so far not been able to match the state-of-the-art accuracy of ANNs or rate-based SNNs. In order to tune the trade-off between rate-based and temporal coding Lagorce et al. (2017) and Sironi et al. (2018) propose to use time surfaces around event input as hierarchical features.

During inference, hidden layers are updated by sending spikes from pre- to postsynaptic neurons. In deep SNNs, simple and efficient models for membrane potential updating and spike generation are typically preferred over more biologically plausible ones (for examples see section 1). One spike from a presynaptic neuron triggers updates of many postsynaptic neurons depending on the number of outgoing connections.

In the output layer of deep SNNs another conversion takes place. Assuming we are dealing with classification tasks, spike trains need to be converted into categories. The simplest form of output code is to report the class corresponding to the neuron with the highest firing rate over some time period or over a fixed number of total output spikes. An extreme case is to report the neuron firing first as the output class, which typically achieves already good performance (Orchard et al., 2015b). However, Diehl et al. (2015) have shown that the classification accuracy increases with the number of output spikes taken into account. Furthermore, SNNs can be specifically optimized to report correct output spikes as early as possible (Neil et al., 2016a). Instead of using the output of single neurons, larger populations of neurons can be used to reduce the variance of the output, or temporal smoothing may be performed.

## 3. Training of deep SNNs

Conventional deep learning is relying on stochastic gradient descent and error backpropagation, which requires differentiable activation functions. Consequently, in order to reduce activations to binary values, often also interpreted as spikes, modifications are required. Such *binary networks* share the discontinuous nature of spikes, but not the asynchronous operation mode with SNNs. The integration of the timing of spikes into the training process, only required for asynchronous SNNs, requires additional effort. Five main strategies for training deep SNNs have been developed over the past years. In this section, we will briefly review these approaches and discuss their advantages and disadvantages:

1. Binarization of ANNs: Conventional DNNs are trained with binary activations, but maintain their synchronous mode of information processing.
2. Conversion from ANNs: Conventional DNNs are trained with backpropagation, and then all analog neurons are converted into spiking ones.
3. Training of constrained networks: Before conversion, conventional DNN training methods are used together with constraints that model the properties of the spiking neuron models.
4. Supervised learning with spikes: Directly training SNNs using variations of error backpropagation.
5. Local learning rules at synapses, such as STDP (Bi and Poo, 1998; Song et al., 2000), are used for more biologically realistic training.

### 3.1. Binary deep neural networks

A simple method to convert ANNs into networks using spikes for communication is to binarize activations for efficient inference (Hubara et al., 2016; Kim and Smaragdis, 2016; Rastegari et al., 2016). Binarized networks propagate information in a synchronized way and layer-by-layer like in conventional DNNs, which does not allow for asynchronous information processing and fast propagation of most salient features as in SNNs (compare Figures 1C,D). However, binarization makes network execution on event-based neuromorphic systems energy-efficient due to sparse activations and computation on demand (see section 4). Furthermore, the computational costs on conventional hardware, like CPUs and GPUs, are also decreased by binarization, since the memory bandwidth as well as the complexity of multiply-add operations are reduced (for a review see Sze et al., 2017), and weight kernels can be re-used within the same network (Hubara et al., 2016). In an extreme case, if both activations and weights are binarized, multiply-add operations can be reduced to bitwise XNORs and bit counting (Rastegari et al., 2016, note that binarized means values in {−1, 1} for this example). In networks with binary activations, weights are usually also quantized to lower-bit representations, and hence, in the following, we review training methods for networks with binary activations and low-bit weights.

To obtain good test performance, binarization of a network (e.g., by rounding) after its training with floating point activations is usually not sufficient (Judd et al., 2015). Instead, networks have to be trained with binarized activations from scratch. Two different approaches are commonly used: training with deterministic and stochastic methods. Deterministic methods usually apply *straight-through-estimators* (Bengio et al., 2013) to approximate non-differentiable activation functions during backpropagation and accumulate gradients on so-called *shadow weights* (Courbariaux et al., 2015). Activations and shadow weights are quantized during the forward pass, while during the backward pass gradients are calculated by assuming that both activations and weights are continuous values. Weight updates are accumulated in shadow weights, which allows quantized weights to change their value in the forward pass in the presence of only very small weight changes during individual training steps. A commonly used stochastic method is *expectation backpropagation*, where neuron activations and synaptic weights are represented by probability distributions updated by backpropagation (Soudry et al., 2014). Esser et al. (2015) adapted this method and showed that ensembles of networks with deterministic binary activations and their ternary weights randomly drawn from the learned probability distributions achieve comparable results to unconstrained networks on classification tasks.

A number of methods exist that improve test performance specifically for networks with binary activations. These methods include normalization of activations (e.g., Hubara et al., 2016), modifications of regularizers (Tang et al., 2017), gradual transitions from soft to hard binarizations (Wu et al., 2015), adding noise on activations and weights (Merolla et al., 2016), and knowledge distillation (Wu et al., 2016; Polino et al., 2018). Nevertheless, networks with binary activations usually show degraded test performance, which can partly be compensated by modifying the network structure, e.g., increasing their width (Mishra et al., 2017). Input and output layers, as well as first and last weight kernels are often not binarized, because only little additional computational resources are needed and otherwise the test performance is likely to significantly drop (e.g., Hubara et al., 2016; Rastegari et al., 2016). Binarization also improves the robustness of networks against adversarial examples (Galloway et al., 2018) and other distortions (Merolla et al., 2016).

Hubara et al. (2016) achieve good results on classification tasks by modifying the binarization scheme of activations *a* from spikes (*a* ∈ {0, 1}) to *a* ∈ {−1, 1}, thereby improving the convergence properties (Li et al., 2017). Although events with payload (here with size 1 bit; see also section 1.1) could be used for communication in order to implement the latter scheme on neuromorphic hardware, communication is not sparse anymore. It is also possible to use payloads with multiple bits per spike to simulate low-precision networks (Courbariaux et al., 2014; Deng et al., 2018), but this approach is outside the scope of the present review.

In summary, binary networks offer efficient inference, which often comes at the cost of slight performance degradations. In addition, the learning process usually takes longer than for unconstrained networks, since the training methods are more complex, intermediate results need to be tracked with floating point precision, and networks are potentially larger. As the need for energy-efficient conventional deep networks increases, binary networks are an active and important research topic independent of their connection to SNNs.

### 3.2. Conversion of deep neural networks

To circumvent the problems of gradient descent in spiking networks, conventionally trained DNNs can be converted into deep SNNs by adapting weights and parameters of the spiking neurons. The goal is to achieve the same input-output mapping with a deep SNN as the original DNN. This mapping, however, does not only include the neural network itself, but also the input- and output-encoding, as discussed in section 2.

Conversion approaches were initially developed to process data from event-based sensors with convolutional networks. Whereas, early attempts used manually programmed convolution kernels on spike train inputs (Serrano-Gotarredona et al., 2009; Pérez-Carrasco et al., 2013) introduced the first systematic way to map conventionally learned CNNs to SNNs. Their conversion approach and that of almost all others follows the idea of rate-coding, such that activations of analog neurons are translated into firing rates of spiking ones. Weights are rescaled according to the parameters of spiking neurons such as leak rates or refractory times. These parameters are not present in conventional CNNs, and need to be set as hyperparameters before conversion. An alternative method is to use the *Neural Engineering Framework* (Eliasmith and Anderson, 2004) to convert restricted Boltzmann machines into spiking networks (Eliasmith et al., 2012). An approach for converting recurrent neural networks under constraints of a neuromorphic platform was presented by Diehl et al. (2016).

The main advantage of the conversion approach is that the full toolkit of deep learning can be exploited, meaning that state-of-the-art deep networks for classification tasks can be straightforwardly converted into SNNs (Hu et al., 2018). For most methods, the original DNNs can be trained without considering the later conversion into SNNs. Once the parameters of the DNN are known, conversion into an SNN usually consists only of parsing and simple transformations, and thus adds only negligible training overhead. Network conversion has set most benchmark records in terms of accuracy for SNNs, with negligible deviations in accuracy from the underlying DNNs (Diehl et al., 2015; Sengupta et al., 2018). It is even possible to provide performance guarantees that can quantify the expected deviations in accuracy (Rueckauer et al., 2017).

Conversion from conventional networks into SNNs, however, comes with its flaws: first of all, not all ANNs can easily be converted into SNNs. One major obstacle is that in ANNs it does not matter if activations are negative, whereas firing rates in SNNs are always positive. In principle, spiking neurons can be divided into excitatory and inhibitory neurons, i.e., neurons with exclusively positive or negative synapses, respectively. However, compared to these biologically realistic SNNs, ANNs can switch the sign of their activation between different inputs. One possible solution, first suggested by Pérez-Carrasco et al. (2013), is to have two spiking neurons for each ANN neuron, one for either positive or negative activations, but mutually exclusive. This problem has gotten less severe with the dominance of rectified linear-unit (ReLU) (Nair and Hinton, 2010) activation functions in deep learning, because then activations are either zero or positive, and can thus easily be translated into firing rates (Cao et al., 2015). Sigmoid activation functions were used in Pérez-Carrasco et al. (2013), but their non-linearity requires additional approximations and introduces additional errors compared to the mainly linear ReLU. Negative activations are a specific problem for softmax-layers at the output, but Rueckauer et al. (2017) presented a practical solution to this problem.

Another limitation of most conversion approaches for CNNs is that max-pooling operations, which are common in state-of-the-art analog deep networks, are difficult to realize in the spiking setting (Yu et al., 2002). The main problem is that the maximum operation is non-linear, and cannot be computed on a spike-by-spike basis. Most approaches (e.g., Cao et al., 2015; Diehl et al., 2015; Sengupta et al., 2018) circumvented this problem by replacing all pooling operations with average pooling, which is easy to implement in SNNs as a linear operation, but leads to a drop of accuracy. A simple mechanism for max-pooling is presented in Rueckauer et al. (2017), where output units contain gating functions that only let spikes from the maximally firing neuron pass, and all other spikes are discarded. This allows a non-linear pooling operation, and contributes to better accuracy of SNNs. Max operations can also be implemented with latency codes (Orchard et al., 2015b), but this is not directly compatible with rate codes typically used for conversion.

While in ReLU networks activations of a layer can be linearly rescaled without changing the final class output by scaling all weights in this layer, SNNs are not immune to such rescaling of weights. Neurons with low firing rates are more susceptible to noisy firing rates and temporal jitter of spikes, which increases the variance of each estimate and elongates the time until a reliable estimate can be formed. Too high firing rates can also be an issue, especially if predicted firing rates exceed the maximum firing rate determined by the neuron parameters. Diehl et al. (2015) improved the performance of deep SNNs significantly by propagating a subset of training examples through the network, observing the firing rates in each layer, and rescaling the input weights to each layer such that a target rate is reached. Rueckauer et al. (2017) and Sengupta et al. (2018) extended this mechanism and improved the results for very deep networks by increasing the robustness against outliers and accounting for the actual firing rates during weight normalization, respectively.

Conversion and weight normalization may come at the cost of more spikes being produced, and thus less energy efficient classification. The trade-off between latency and accuracy in SNNs (Diehl et al., 2015) allows compensating this effect by training deep SNNs to achieve a target performance level as early as possible (Neil et al., 2016a). Nevertheless, ANNs converted into SNNs using rate codes are in general not particularly efficient in terms of spikes being produced, because multiple spikes are necessary to represent one real-valued activation. In the worst case the SNN might need more spike operations than the ANN needs multiply-adds. However, spike operations are cheaper than real-valued matrix multiplications, and can be implemented on very efficient neuromorphic hardware (section 4).

In order to address the inefficiencies of conversion approaches based on rate codes, an important direction of research investigates the use of alternative spike codes based on the timing information. This is particularly important when inputs come from event-based sensors, and therefore naturally contain precise timing information. The HFirst model (Orchard et al., 2015b) introduced a spiking adaptation of an HMAX hierarchical network (Riesenhuber and Poggio, 1999) with predefined Gabor filters. A temporal winner-take-all mechanism replaces the computation of rate maxima, and thus simplifies the classification. Time surface features (Lagorce et al., 2017) capture the local spatio-temporal dynamics around events by computing a continuous-valued feature for small spatial and temporal windows around each event, which describes the spiking activity of nearby events. The HATS method (Sironi et al., 2018) divides the image into a regular grid of cells, and smoothes all time surfaces within each cell and specified time window. A histogram of averaged time surfaces is formed, and the resulting feature vector is fed to a standard classifier, such as an SVM or a neural network. Approaches such as HATS are tailored for neuromorphic vision sensors, and have no equivalent in conventional deep learning or computer vision. Zambrano and Bohte (2016) introduces an asynchronous pulsed Sigma-Delta coding scheme for SNNs, which maintains the accuracy of the underlying ANN, but utilizes far fewer spikes than other ANN-to-SNN conversion methods.

### 3.3. Training of constrained networks

Whereas the conversion approaches presented in section 3.2 start from fully trained ANNs, and then convert these networks into SNNs, Esser et al. (2015) coined the term *constrain-then-train* for approaches that include constraints due to the properties of spiking neurons or the target hardware already during the training process. Conventional learning rules for ANNs, such as backpropagation, are applied to learn the optimal weights under constraints of the spiking model. After training, a conversion into an SNN is performed, where the parameters of the constrained ANN model are directly used as parameters of the SNN without further weight scaling. There is a fine line between conversion and constrain-then-train methods, since conversion algorithms also put some constraints on the ANN model, e.g., they demand the use of ReLU activation functions or zero biases, and constrain-then-train models also convert ANNs into SNNs. The main difference is that conversion methods train the ANN just once and then map the weights for arbitrarily specified parameters of spiking neurons, whereas for constrain-then-train methods the ANN is trained for one specific setting of spiking neuron model parameters. If later these spiking neuron parameters should change then a complete retraining of the constrained ANN is required, which is not necessary for conversion methods. Constrain-then-train models have the potential to adapt better to the target platform than converted models, because the ANN training already considers specifics of the final SNN. As a result, constrain-then-train methods often yield better accuracy than generic conversion methods, at the expense of more complicated ANN training.

Constrain-then-train methods need to transform spiking neuron models into a continuous-valued and differentiable form that can be trained via backpropagation. In Esser et al. (2015) a training network was introduced which used continuous valued weights and activations constrained to be in the range [0, 1]. Such a network after training yields values representing probabilities of spikes occurring or binary synapses being on. The learned probabilities are used to generate samples of deployment networks with low-bit synapses, matching the constraints of the TrueNorth target platform (Merolla et al., 2014). This results in highly accurate classifiers for MNIST, at very low energy costs (see also section 4). This approach was improved and extended to multi-chip setups in Esser et al. (2016).

For more realistic models of spiking neurons it is often possible to derive or approximate a transfer function that relates a constant input current and neuron parameters (e.g., refractory period, reset voltage, etc.) to an average firing rate (Gerstner and Kistler, 2002; Burkitt, 2006). This will typically result in a non-differentiable function, because output spikes will only be generated after the input current crossed a threshold. Instead of ignoring the more complex activation functions of spiking neurons and simply using ReLUs, constrain-then-train approaches typically introduce various smoothing approximations to model spike generation and the variability of spike times and rates more accurately (O'Connor et al., 2013; Hunsberger and Eliasmith, 2016). The key idea is modeling this variability, e.g., noisy firing rates and jitter on spike times, in the input spike trains to obtain a differentiable activation function that enable training with backpropagation. After training, all neurons are turned into spiking ones using the model parameters set before training and the newly learned weights. The goal is to have the rate-coded SNN perform similar to the ANN resulting from the constrained learning process.

One of the first successful applications of deep SNNs was presented by O'Connor et al. (2013), who used the so-called *Siegert approximation* (Jug et al., 2012) to train a spiking DBN. Their training did not involve backpropagation, but contrastive divergence (CD) learning (Hinton et al., 2006). However, the key ideas remain the same. For CD learning in SNNs, firing rates are used as proxies for activation probabilities that are normalized into the range [0, 1]. This transformation can be easily achieved by assuming that the inverse of the refractory period yields the maximum firing rate, and therefore also the maximum activation during training with CD. The different layers of the DBN are trained sequentially, and after training all neurons in the network are converted into spiking neurons, using the parameters defined before or during training. The Siegert approximation provides more accurate predictions of the output firing rate of leaky integrate-and-fire (I&F) neurons, if the inputs are Poisson spike trains, rather than constant currents. A conventional DBN trained with Siegert approximation can thus be converted almost loss-less into a spiking DBN. Interestingly, the DBN trained on conventional MNIST also performs well when being fed inputs from an event-based vision sensor recording MNIST digits. Despite the mismatch to the assumed Poisson distribution, visual recognition with high accuracy can be performed in real-time.

A similar concept was used in Hunsberger and Eliasmith (2015), where a spiking CNN was trained with backpropagation, using a so-called *soft leaky I*&*F*. This neuron model employs a smoothed and therefore differentiable version of the I&F transfer function, which can be used for gradient descent, and additionally adds noise to the training process. Hunsberger and Eliasmith (2016) show competitive results with this approach on a number of benchmarks, including the challenging ImageNet dataset.

### 3.4. Supervised learning with spikes

Whereas constrain-then-train methods reduce the training of SNNs to training methods of conventional ANNs, various approaches have been proposed that directly introduce supervised learning on the level of spikes. These approaches do not necessarily aim for biological plausibility, which is the goal of approaches using local learning with STDP, as discussed in section 3.5. Instead, supervised training methods with spikes typically use variants of backpropagation to train deep SNNs. The obvious advantage of spike-based learning rules for SNNs is that they are not constrained to mean-rate codes, but can learn to utilize spatio-temporal patterns in spike trains, which can arise in inputs from event-based sensors. This might come at the cost of longer training times, because fully spiking simulations are computationally more expensive than simulations of conventional DNNs, although the number of spikes needed is typically lower than in rate-coded simulations of SNNs.

There are several supervised learning methods for spiking networks that work only for single layers, such as ReSuMe (Ponulak and Kasiński, 2010) or the Tempotron (Gütig and Sompolinsky, 2006). The focus of this review is on deep spiking networks, so we discuss in the following only methods that implement some form of backpropagation to train multiple layers. Another important distinction is the nature of the target signal: whereas some methods (e.g., Bohte et al., 2002) require a target spike train and during training try to reproduce the temporal pattern for a given input, for most other methods presented here (e.g., Lee et al., 2016) it is sufficient if a target label is provided. The training goal can then either be to have the correct output neuron firing more than all others, or having the correct output neuron firing first (e.g., Mostafa, 2018). Intermediate forms, such as defining a regular target spike train for the correct class as in Kulkarni and Rajendran (2018) are also possible.

The key for many spike-based learning rules for multi-layer SNNs is to find a real-valued and almost-everywhere differentiable proxy, on which backpropagation can be performed. The earliest attempts at training multi-layer SNNs fall into this category, most notably SpikeProp (Bohte et al., 2002) and variants (Schrauwen and Van Campenhout, 2004; McKennoch et al., 2006). SpikeProp derives a backpropagation rule for spike times in the output layer, and Booij and tat Nguyen (2005) showed an extension to patterns of multiple spikes. However, SpikeProp has not been applied to problems at the scale of modern deep learning applications, yet, because this method is computationally expensive.

Recently, Lee et al. (2016) have introduced a spike-based backpropagation rule that can train deep SNNs for conventional classification tasks (given only labels, but not target spike trains) directly from spike signals. The key trick is to perform stochastic gradient descent on real-valued membrane potentials. Discontinuities at the times of spikes are handled via low-pass filtering before they are used for backpropagation. Together with a variety of optimizations, this method achieves state-of-the-art results for deep SNNs on tasks such as MNIST, and its event-based counterpart N-MNIST (Orchard et al., 2015a). (Kulkarni and Rajendran, 2018) follow a similar approach and achieved similar results, but minimized the distance between network output and a regular firing target spike train for the desired output neuron, instead of the squared distance between normalized network output and one-hot labels.

O'Connor and Welling (2016) propose a spiking network that approximates a deep MLP with ReLU activations using signed spikes. During training backpropagation operates on collected spike statistics. Similarly, Stromatias et al. (2017) bin spike trains, and fine-tune output layers of deep SNNs by performing gradient descent on these real-valued histogram bins. A different approach is taken in Mostafa (2018), where the time of first spike for each neuron is used as its activation value during training. This results in sparsely firing SNNs that are able to utilize temporal patterns in input sequences. A spatio-temporal backpropagation rule for SNNs is derived in Wu et al. (2017). The authors are separating spatial input signals, which come from other neurons, from temporal dynamics arising from the spiking behavior of the neuron itself. Their results consistently show improvements over methods unaware of the temporal aspects. An interesting hybrid model has recently been proposed by Jin et al. (2018), which uses a backpropagation rule for a rate-coded error signal on a longer "macro" time-scale, and combines this with an update on a shorter "micro" time-scale which captures individual spike effects. The method leads to state-of-the-art results on static MNIST and N-MNIST (Orchard et al., 2015a).

Overall, the past years have clearly yielded an increasing number of spike-based learning approaches for supervised training, occasionally outperforming approaches based on conversion alone. Their benefits for machine learning tasks on neuromorphic sensor data is still not fully explored, but potentially even greater performance gains could be achieved by exploiting temporal codes that deviate from pure rate models.

### 3.5. Local learning rules

It is of great interest for neuroscience to understand how hierarchically organized neural networks can be trained with local learning rules such as STDP (Markram et al., 1997; Bi and Poo, 1998) or Hebbian learning (Hebb, 1949). For practical applications, the use of local learning rules is very attractive, because it would allow very hardware-efficient ways of training DNNs. In addition, spike-timing dependent learning allows detecting spatio-temporal patterns as features.

The main obstacle for the use of purely local learning rules in deep networks is the difficulty to perform backpropagation of supervised error signals. An error signal might only be available at the output layer, and since the information flow in biological axons is assumed to be uni-directional, it is unclear how error information can reach lower levels of the hierarchy at all. Typical feed-forward architectures used in machine learning are not capable of providing the necessary training information to synapses learning with local rules. Hence, most of the following studies investigating local learning in hierarchies introduce recurrent feedback connections that modulate learning in lower layers.

One insight from biology is that feedback connections, i.e., connections projecting from higher to lower layers, are common and important in hierarchically organized networks for information processing, such as the cortex (Markov et al., 2014). Furthermore, feedback connections could provide training signals in the framework of *predictive coding* (Rao and Ballard, 1999). Another interesting perspective is that random back-projections of error signals are sufficient to train lower layers (Lillicrap et al., 2016). This concept has been recently demonstrated for deep SNNs (Neftci et al., 2017), and for networks with spiking multi-compartment neurons (Guerguiev et al., 2017). Although the performance does not match that of conventional machine learning techniques (Bartunov et al., 2018), this approach is a proof-of-concept that biologically plausible training of deep SNNs with local learning rules is possible. In particular, random backpropagation is one possible solution for training spiking and biologically realistic networks with backpropagation, despite deviating from the requirements of classical backpropagation, namely precise calculation of real-valued gradients, a separation and synchronization of forward- and backward-passes, and symmetry of weights in both directions (Bengio et al., 2015a). Mostafa et al. (2017b) show how feature hierarchies can be trained with local errors from random auxiliary classifiers, and how training can work despite the asynchronous updates found in SNNs. Another recent line of research has established links between inference in energy-based networks and backpropagation (Bengio et al., 2017). A proposal for solving the credit assignment problem by using multiple layers with local rules was made, and it was shown that early steps of inference in this iterative method in autoencoder-like models yield activation changes in hidden layers that approximate backpropagation. Furthermore, the required updates are compatible with STDP, and could thus be implemented in a biologically plausible way (Bengio et al., 2015b).

In general, the function of STDP is highly dependent on the network architecture, in which it is applied. In competitive networks, STDP is capable of solving unsupervised learning tasks such as clustering (Masquelier et al., 2009; Nessler et al., 2013). This is encouraging, since recent work (e.g., Coates et al., 2011; Dundar et al., 2015) has shown that competitive convolutional networks can be trained with unsupervised learning of filters. If spiking neurons are connected according to the structure of restricted Boltzmann machines, contrastive divergence can be approximated in an event-based fashion (Neftci et al., 2014, 2016). In principle, this can be extended to multi-layer DBNs, but then training occurs only within each layer. Spiking CNNs (Kheradpisheh et al., 2018; Lee et al., 2018) and autoencoders (Panda and Roy, 2016) can be trained layer-by-layer with unsupervised STDP, assuming that weight updates for identical kernels are shared between their applications to different spatial locations, and single-layer supervised frame-based learning is used for the output layer (Stromatias et al., 2017). Mozafari et al. (2018) add multiple layers with reward-modulated STDP to such networks to obtain fully spiking supervised training. Recently, in order to simultaneously train all layers with STDP within a deep network Thiele et al. (2018) introduced neurons with two integrate-and-fire units decoupling learning with STDP and inference. These approaches discussed above, however, do not reach the accuracy of conventional deep neural networks trained with backpropagation.

## 4. Neuromorphic hardware

There is a big discrepancy between the promise of efficient computing with SNNs and the actual implementation on currently available computing hardware. Simulating SNNs on von Neumann machines is typically inefficient, since asynchronous network activity leads to a quasi-random access of synaptic weights in time and space. Ideally, each spiking neuron is an own processor in a network without central clock, which is the design principle of *neuromorphic* platforms. The highly parallel structure, sparse communication, and in-memory computation proposed by SNNs stands in contrast to the sequential and central processing of data constrained by the memory wall between processor and memory on CPUs and GPUs. The computational efficiency of SNNs can be observed in brains that can solve complex tasks while requiring less power than a dim light bulb (Laughlin and Sejnowski, 2003). To close the gap in terms of energy-efficiency between simulations of SNNs and biological SNNs in the last decade several neuromorphic hardware systems were developed which are optimized for execution of SNNs (see Table 1; for a review of technical specifications see Furber, 2016; Singh Thakur et al., 2018). The energy-efficiency of neuromorphic systems makes them ideal candidates for embedded devices subject to power constraints, e.g., mobile phones, mobile and aerial robots, and internet of things (IoT) devices. Furthermore, neuromorphic devices could be utilized in data centers to reduce the cost of cloud applications relying on neural networks.

**Table 1:** This table lists built neuromorphic systems for which results with deep SNNs on classification tasks have been shown (for extended lists of hardware systems that may potentially be used for deep SNNs see, e.g., Indiveri and Liu, 2015; Liu et al., 2016).

	**System overview**		**On-chip learning**	**Inference of deep SNNs**					
**Name**	**Type of hardware**	**Neuron and synapse count**	**Plasticity rules**	**Network type**	**Network size**	**Dataset**	**Test accuracy**	**Throughput (in img/s)**	**Energy consumption (per image)**
TrueNorth (Merolla et al., 2014)	digital	single chip: 4096 cores, 1M neurons, 256M synapses; up to 8 chips	None	Deep CNNs: (a, b) Esser et al. (2015) (c) Esser et al. (2016)	(a) 1920 cores (b) 5 cores (c) 8 chips	(a, b) MNIST (c) CIFAR10 and many more	(a) 99.4% (b) 92.7% (c) 89.3%	(a, b) 1000 (c) 1249	(a) 108 μJ (b) 0.268 μJ (c) 164 μJ
SpiNNaker (Furber et al., 2013)	digital	single chip: 18 ARM cores, approx. 1k neurons and 1k synapses per core for real-time simulations; up to 576 chips	flexible, e.g., unsupervised (Jin et al., 2010) and supervised (Mikaitis et al., 2018) STDP	DBN: 2 hidden layers with 500 neurons each (Stromatias et al., 2015)	1 chip	MNIST	95%	91	3.3 mJ
BrainScaleS (Schemmel et al., 2010; Brüderle et al., 2011)	mixed-signal	wafer with 384 cores, 200k neurons, 45M synapses	STDP (Schemmel et al., 2006; Pfeil et al., 2013b)	MLP: 2 hidden layers with 15 neurons each (Schmitt et al., 2017)	14 cores	downscaled MNIST	95%	10000	7.3 mJ

*Spike communication in all of these systems is asynchronous. The SpiNNaker system is the only system listed in this table that allows events with payload (see section 1). Note that none of these systems natively support batching of inputs as commonly used in conventional deep learning*.

Inspired by biology, neuromorphic devices share the locality of data to reduce on-chip traffic, mostly reflected by using spikes for communication and limiting the fan-in of neurons. The massive parallelism of neuromorphic devices manifests itself in the physical representation of neurons and synapses on hardware inspired by the seminal study of Mead (1990) (for a review, see Indiveri et al., 2011). Analog neuromorphic systems, which implement functionalities of spiking neurons and synapses with analog electronic circuits, usually have a one-to-one mapping between neurons and synapses in the network description and on hardware. In contrast, digital systems implement the parallel structure less fine-grained by grouping and virtualizing neurons on *cores* (hundreds for the TrueNorth and thousands for the SpiNNaker system, see also Table 1). However, compared to the extensive virtualization on CPUs and GPUs, i.e., the total number of neurons in a network divided by the number of cores, the virtualization on neuromorphic systems is rather low. This leads to less flexibility in terms of connectivity and size of networks, and thus hardware demonstrations that show functional deep SNNs are few. All hardware systems listed in Table 1 share an asynchronous computation scheme that enables computation on demand and reduces power consumption in case of low network activity.

In principle, neuromorphic hardware could be used for both training and inference of SNNs. While original and constrained DNNs (section 3.3) can usually be trained on GPUs and are then converted to SNNs (section 3.2), spike-based training (section 3.4) and especially local learning rules (section 3.5) are computationally more expensive on von Neumann machines and, hence, could highly benefit from hardware acceleration. However, so far, spike-based training and local learning rules have not been shown for competitive deep networks. Rapid developments in this area of research makes it difficult to build dedicated hardware for training, since their design and production is time-consuming and costly (see also section 6).

### 4.1. Inference on neuromorphic hardware

Once the parameters of SNNs are obtained by any of the training methods reviewed in section 3, usually these networks have to be adapted to the specific hardware system to be used for inference. Analog neuromorphic systems suffering from parameter variation may require cumbersome fine-tuning of pre-trained networks with the hardware system in-the-loop (Schmitt et al., 2017). This is not always practical, because the re-configuration of neuromorphic systems is often slow compared to, for example, CPUs and GPUs. Another common approach to improve the test performance is to incorporate hardware constraints like, for example, limited counts of incoming connections and quantized weights into the training process (section 3.3). Once parameters are trained and the device is configured, inference is usually fast and energy-efficient due to their optimization for spike input and output. To our knowledge only for the TrueNorth, SpiNNaker and BrainScaleS hardware system results were shown, in which deep SNNs on silicon chips were used for classification tasks with the complexity of at least MNIST (for hardware specifications and classification performances, see Table 1). For other promising neuromorphic systems no results for deep SNNs are shown yet (Park et al., 2014; Lin et al., 2018), or the presented neuron and synapse count is too small to show competitive results (Pfeil et al., 2013a; Schmuker et al., 2014; Indiveri et al., 2015; Qiao et al., 2015; Moradi et al., 2017; Petrovici et al., 2017). Prototypical software implementations and field-programmable gate array (FPGA) systems are not considered in this study. As an exception, we would like to mention the novel Intel Loihi chip (Davies et al., 2018), for which results of a single layer network on preprocessed MNIST images on a prototypical FPGA implementation are shown (Lin et al., 2018). Once commissioned, Loihi's large number of neurons, their connectivity and configurability, and on-chip learning capabilities could be a good basis to enable deep networks on raw image data. Table 1 shows deep SNNs on the SpiNNaker and BrainScaleS systems that approximate multi-layer perceptrons (MLPs) and rate-based deep belief networks (DBNs), respectively, showing network activity like exemplarily plotted in Figure 1D. In contrast, deep CNNs are binarized for their execution on the TrueNorth system (compare to Figure 1C). This means that neuron activations on TrueNorth are represented by single spikes and each neuron in a network is stateless and fires at most once for each input. In other words, spikes do not contain temporal information anymore, but the high throughput makes inference energy-efficient.

Are the presented neuromorphic systems more power-efficient than GPUs? The answer to this question very much depends on the chosen benchmark task, and we can give only approximate numbers for frame-based classification tasks (for further discussions see section 6). Since power measurements on modern mobile GPUs (Nvidia Tegra X1) are only reported for large networks (AlexNet) on comparably large images from the ImageNet dataset (NVIDIA Corporation, 2015), and power numbers of the most efficient neuromorphic system are recorded for custom networks on smaller images from the CIFAR10 dataset (Esser et al., 2016), a straight-forward comparison is not possible. However, if we assume a linear decrease in the number of operations with the area of the input image, which is approximately true for convolutional networks, the reported energy of 76 mJ for GPUs to process an image of size 224 × 224 scales down to approximately 2 mJ for an image from the CIFAR10 dataset with size 32 × 32. This energy consumption is approximately one order of magnitude higher than for the most power-efficient neuromorphic solution, i.e., binarized DNNs on the TrueNorth system (for numbers see Table 1). Since the energy consumption of most neuromorphic systems is dominated by that of synaptic events, i.e., communication and processing of spikes, higher benefits are expected for models that exploit sparse temporal codes, rather than rate-based models.

### 4.2. On-chip learning

Although unified methods to train SNNs are still missing, the SpiNNaker and BrainScaleS hardware systems implement spike-timing-dependent plasticity (STDP), a local unsupervised learning rule inspired by biology. Synaptic weights are updated by means of local correlations between pre- and postsynaptic activity (see also section 3.5). Neuromorphic systems are valuable tools to investigate such local learning rules, because the training of networks with STDP often requires long simulations of SNNs in terms of biological time, and neuromorphic systems usually accelerate such simulations compared to conventional computers. The BrainScaleS system (Schemmel et al., 2010) and its successor (Aamir et al., 2018) is an especially promising candidate for on-chip learning due to its acceleration of up to a factor of 10000 compared to biological real time, but so far STDP is only shown for small networks on a prototype chip (Pfeil et al., 2013b) and shows significant parameter variation due to imperfections in the production process (Pfeil et al., 2012). In addition, Friedmann et al. (2017) investigated the integration of on-chip plasticity processors into the BrainScaleS system to modulate STDP based on the model of neuromodulators in biology (Pawlak et al., 2010) allowing for supervised training. Although the implementation of STDP is in terms of chip area costly for the presented neuromorphic systems, novel electronic components, so called memristors, may allow for much higher densities of plastic synapses (Jo et al., 2010; Saïghi et al., 2015; Boyn et al., 2017; Burr et al., 2017; Pedretti et al., 2017).

## 5. Applications

With the current big success of deep learning in conventional machine learning it is tempting to view deep SNNs exclusively as a more efficient replacement of conventional DNNs. This view is reflected in the way deep SNNs are benchmarked against conventional machine learning approaches by their classification accuracy on standard datasets such as MNIST or CIFAR. Such comparisons are certainly important, because they show that SNNs can be powerful classifiers in the classical machine learning setup. However, entirely focusing on accuracy can easily become misleading when it comes to the potential advantages of SNNs, namely efficiency and low latency. Achieving state-of-the-art accuracy with rate-based networks often comes at the cost of having very high firing rates and long integration times to obtain reliable results (Rueckauer et al., 2017).

Using temporal codes (Mostafa et al., 2017a) is an attractive alternative, but, so far, such approaches have not reached state-of-the-art accuracy. The ability to utilize the information of precise timing is a feature than only SNNs - whether deep or not - offer, but which we think has not been investigated and exploited enough. Temporal codes allow to represent features of the input in precise spike patterns of small groups of neurons (Gütig, 2014), and there is no more need to precisely estimate firing rates first (Gardner et al., 2015). Information in SNNs might also be encoded in spike times relative to background oscillations, which has been shown to benefit learning in recurrent networks (Neil et al., 2016b), and allows to encode multiple features in parallel (Kayser et al., 2009). The use of precise timing information always carries the risk of being susceptible to noise and temporal jitter, but the approaches mentioned above exhibit quite high degrees of noise robustness. Several promising, but not necessarily deep, temporal coding and learning schemes have been proposed (e.g., Gütig and Sompolinsky, 2006; Tapson et al., 2013; Lagorce and Benosman, 2015), but no applications at the scale that conventional deep learning is addressing have been demonstrated, yet.

The greatest impact of deep SNNs is expected in the processing of inputs from event-based sensors, because only SNNs are able to fully exploit the precise temporal information such sensors offer. To stimulate research in this direction, we and others from the neuromorphic engineering community have argued that new benchmarks are necessary, which do not carry the legacy of evaluation in conventional machine learning or computer vision (e.g., Iyer et al., 2018). Instead, they should be specifically designed to show the advantages of the neuromorphic approach (Orchard et al., 2015a; Tan et al., 2015; Barranco et al., 2016; Hu et al., 2016; Liu et al., 2016; Binas et al., 2017; Mueggler et al., 2017). Such datasets have only recently become available, but already had a beneficial effect on the fair comparison between different SNN approaches. Although this is a step into the right direction, it is still problematic that most of these datasets are event-based variants of conventional classification tasks, such as MNIST digits recorded with a dynamic vision sensor. In order to fully exploit the strengths of the neuromorphic approach, we suggest that a careful analysis of use cases is necessary. We propose that there are at least two classes of use cases that should be investigated deeper: First, the case where neuromorphic sensors provide additional features (e.g., precise timing and low latency) that can be exploited by a deep SNN. Second the case where low-power and low-latency aspects of deep SNNs really make a difference in real-world applications.

We are currently observing the interesting trend that event-based vision becomes increasingly interesting for research communities rooted in classical computer vision and robotics. Advantages of using event-based sensors have been demonstrated for diverse applications such as tracking (Mueggler et al., 2014; Lagorce et al., 2015; Gallego et al., 2018), stereo vision (Rogister et al., 2012; Osswald et al., 2017; Martel et al., 2018), optical flow estimation (Benosman et al., 2014; Bardow et al., 2016), gesture recognition (Lee et al., 2014; Amir et al., 2017), scene reconstruction (Carneiro et al., 2013; Kim et al., 2016; Rebecq et al., 2017), or SLAM (Weikersdorfer et al., 2014; Vidal et al., 2018). All of these applications benefit from the high speed and the high dynamic range of spike-based sensors to solve tasks, such as high-speed localization and navigation, which are very hard with conventional vision sensors. However, only few of these approaches use SNNs for event-based post-processing, or run on neuromorphic hardware. A notable exception is the gesture recognition system in Amir et al. (2017), which was designed to highlight the benefits of combining a dynamic vision sensor with the TrueNorth processing chip. We think there is great potential for fully event-based sensing and processing systems, and given the success of conventional deep learning, deep SNNs on neuromorphic hardware platforms seem like an obvious choice. Initial demonstrations on simpler classification tasks are encouraging (Orchard et al., 2013; Merolla et al., 2014; Neil and Liu, 2014; Stromatias et al., 2015), but more research is needed to create deep SNNs specifically tailored for event-based sensor input.

Conventional machine learning has realized that a co-development of algorithms and hardware is necessary by moving toward low-bit precision or binary networks. The same is true in the neuromorphic domain, and further adaptations of vision sensors to the capabilities of post-processing systems have a great potential. Once the performance of deep SNNs is good enough, neuromorphic hardware implementations could become the method of choice for applications, wherever low power is of particular importance. Besides battery powered robots and embedded devices, a particularly interesting application field is brain-machine interfaces (Dethier et al., 2013; Corradi and Indiveri, 2015; Boi et al., 2016), where small size, low energy consumption, low heat dissipation, robustness, and the ability to decode in real-time are important. The fact that SNNs can process recorded biological spikes without further transformation adds to the appeal of such systems. The field of automated driving is expected to become another major application area, where the focus is less on low power, but on enhancing safety critical functions by exploiting speed and dynamic range of neuromorphic sensors (Binas et al., 2017; Sironi et al., 2018). On-board, SNNs can process sensor information in real-time, potentially improving emergency brake assistants, which have to deal with challenging light conditions as well as suddenly appearing road users, or providing reliable perception during evasive high-speed maneuvers.

Finally, deep SNNs and their hardware implementations will continue to be used as models of computation in biological neural circuits, and thus form a valuable tool for Computational Neuroscience (Singh Thakur et al., 2018). Hardware platforms such as SpiNNaker (Furber et al., 2014), Neurogrid (Benjamin et al., 2014), TrueNorth (Merolla et al., 2014), and BrainScaleS (Schemmel et al., 2010) have shown great potential to accelerate large-scale brain simulations. Recently discovered analogies of real neural representations in the cortex to those learned in deep networks (Kriegeskorte, 2015; Yamins and DiCarlo, 2016) have increased the interest of the neuroscience community in deep learning, and deep SNNs could become an interesting tool to study the interplay of neuronal structure, plasticity, and spiking dynamics in large-scale simulations.

## 6. Discussion

Advances in deep SNNs have helped closing the performance gap to conventional DNNs. However, the promise of low-power inference is not fulfilled yet, since network conversion (section 3.2) and training of constrained networks (section 3.3) result in spike-based networks that encode information mostly in their neurons' mean firing rates, but do not exploit the potential of encoding information in the timing of single spikes. Although these networks achieve a remarkable performance on various benchmark datasets, the average firing rates of their neurons are comparably high for static input images, and hence their energy-efficiency on neuromorphic systems is not significantly better than for conventional DNNs on GPUs (section 4). To reduce firing rates and increase energy-efficiency spike-based training methods (section 3.4) and local learning rules (section 3.5) have become increasingly popular research topics. Their accuracy on machine learning benchmarks is not quite at the level of converted networks, but recent approaches by Lee et al. (2016) or Jin et al. (2018) could partly close the gap. Besides, the choice of benchmarks that usually consist of frame-based images converted to spiking representations (section 2) puts spike-based rules at a disadvantage. Finding local learning rules that can achieve the same performance as backpropagation would be a result with great implications beyond machine learning applications, since it could possibly explain how brains can learn their remarkable capabilities with the constraints for information and error signal routing imposed by biology (Bengio et al., 2015a).

We have argued in section 5 that further opportunities for deep SNNs will arise when appropriate benchmark datasets recorded with event-based sensors become available. The rise of deep learning has largely been driven by the availability of large common benchmarks such as ImageNet (Russakovsky et al., 2015). Similarly large and challenging neuromorphic datasets are not available, yet, but we see a positive trend and increased awareness of the community. First benchmarks for real-world applications in automated driving (Binas et al., 2017; Sironi et al., 2018) and robotics (Mueggler et al., 2017) have been released, and together with convincing results on problems where conventional systems struggle (Kim et al., 2016; Vidal et al., 2018), we expect that this will lead to increasing demand for efficient event-based post-processing systems. Fully event-based systems are not only energy-efficient, but could also better exploit the rich temporal dynamics of the real world than frame-based approaches, which artificially introduce time steps through sensing or processing components. For agents interacting with the real world, temporal information on different time-scales plays an important role, because critical situations require short reaction times hardly accessible by frame-based perception. Deep SNNs have the important property of providing good early estimates, which improve when given more processing time (see Figure 1F). Although mechanisms to provide early estimations are also proposed for conventional DNNs (e.g., Teerapittayanon et al., 2016), their implementations are rather artificial and not as seamlessly integrated as in SNNs. Fischer et al. (2018) proposed a hybrid solution between conventional DNNs and deep SNNs, called *streaming rollouts*, which are conventional synchronous DNNs that share a dense temporal integration and fast response times with deep SNNs.

A future direction of research may be the incorporation of recurrence into deep SNNs improving the storage and integration of temporal information. Recurrent SNNs have shown remarkable performance in sequence recognition (Zhang et al., 2015; Panda and Srinivasa, 2018) and generation tasks (Rajan et al., 2016; Panda and Roy, 2017). In these cases, instead of a deep or structured recurrent architecture the networks were configured as *liquid state machines* (Maass et al., 2002), which consist of a reservoir of randomly and recurrently connected neurons, followed by a linear readout. Recent work (e.g., Diehl et al., 2016; Bellec et al., 2018) have shown how standard recurrent network architectures such as long short-term memory networks (LSTMs, Hochreiter and Schmidhuber, 1997) can be ported into the spiking domain, whereas Neil et al. (2016b) have shown a way to process event-based data with otherwise standard recurrent networks. Combining recurrent architectures with the intrinsic short-time memory of spiking neurons appears as a promising route for efficiently solving real-world pattern recognition tasks.

As deep SNNs become larger and capable of solving tasks that are more complex, training time is likely to become a bottleneck due to the more complex training methods compared to conventional DNNs, as well as inefficient spiking simulations on conventional computing platforms. It is therefore important to advance neuromorphic hardware systems for large-scale deep SNNs, and not only consider energy-efficient inference, but also training. Efficient training can be either realized via on-chip learning rules like STDP as discussed in section 4.2, by using neuromorphic systems in-the-loop, i.e., computing weight updates on the host computer and then re-configuring the hardware system, or by hybrid solutions. However, contemporary neuromorphic systems share a comparably low bandwidth to the host computer, usually sufficient for spike input and output, but inappropriate for a frequent re-configuration of the device. This is why the development and investigation of hierarchies of learning rules both on algorithmic and hardware level, ranging from in-memory plasticity rules like STDP to global reward signals, would be a valuable topic for future studies. Friedmann et al. (2017) and Lin et al. (2018) already proposed architectures that go into this direction, and it will be interesting to see first large-scale experimental results and further developments in the near future. Although such systems may allow for the exploration of networks with a size and complexity not accessible with current hardware systems, their development is time consuming, costly, and will most likely not offer the flexibility to catch up with the latest algorithmic developments. Compared to digital systems, analog systems promise a higher energy-efficiency. However, the training of analog systems requires additional efforts (see section 4.1) and the short- and long-term variations in their parameters and computations, e.g., caused by temperature fluctuations, pose great challenges.

## Author contributions

All authors listed have made a substantial, direct and intellectual contribution to the work, and approved it for publication.

### Conflict of interest statement

The authors declare that the research was conducted in the absence of any commercial or financial relationships that could be construed as a potential conflict of interest.
